# Response of monoflagellate pullers to a shearing flow: A simulation study of microswimmer guidance

**Published:** 2018-12-26

**Authors:** Benjamin J. Walker, Kenta Ishimoto, Richard J. Wheeler, Eamonn A. Gaffney

**Affiliations:** 1 Wolfson Centre for Mathematical Biology, Mathematical Institute, University of Oxford, Oxford OX2 6GG, United Kingdom; 2 Graduate School of Mathematical Sciences, The University of Tokyo, Tokyo 153-8914, Japan; 3 Sir William Dunn School of Pathology, University of Oxford, Oxford OX1 3RE, United Kingdom; 4 Nuffield Department of Medicine, University of Oxford, Oxford OX3 7BN, United Kingdom

## Abstract

Microscale swimming may be intuited to be dominated by background flows, sweeping away any untethered bodies with the prevalent flow direction. However, it has been observed that many microswimmers utilize ambient flows as guidance cues, in some cases resulting in net motion upstream, contrary to the dominant background fluid direction and our accompanying intuition. Thus the hydrodynamic response of small-scale motile organisms requires careful analysis of the complex interaction between swimmer and environment. Here we investigate the effects of a Newtonian shear flow on monoflagellated swimmers with specified body symmetry, representing, for instance, the *Leishmania mexicana* promastigote, a parasitic hydrodynamic puller that inhabits the microenvironment of a sandfly vector midgut and is the cause of a major and neglected human tropical disease. We observe that a lack of symmetry-breaking cellular geometry results in the periodic tumbling of swimmers in the bulk, with the rotations exhibiting a linear response to changes in shearing rate enabling analytic approximation. In order to draw comparisons with the better-studied pushers, we additionally consider virtual *Leishmania* promastigotes in a confined but typical geometry, that of a no-slip planar solid boundary, and note that in general stable guided taxis is not exhibited amongst the range of observed behaviors. However, a repulsive boundary gives rise to significant continued taxis in the presence of shearing flow, a phenomenon that may be of particular pertinence to the infective lifecycle stage of such swimmers subject to the assumption of a Newtonian medium. We finally propose a viable and general *in vitro* method of controlling microswimmer boundary accumulation using temporally evolving background shear flows, based on the analysis of phase-averaged dynamics and verified *in silico*.

## I Introduction

In the microscale world of unicellular swimmers, the background dynamics of the environment may seem to dominate cell motion. However, such motile microorganisms are guided by background flows, enabling the directed taxis of cells in their microenvironment. For example, a background shear flow is seen to influence the swimming direction of mammalian spermatozoa near a boundary [1–3], and bias the orientation of *Escherichia coli* both in the proximity of a planar boundary and in the bulk fluid [4,5]. Such a directed response to background flow, known as rheotaxis, is hypothesized in cases to be a contributing factor to the function or survival of the swimmer, as in the case of the canonical human spermatozoon [2,6]. Rheotaxis is widely prevalent in nature across a range of scales, being commonly observed in fish [7] and additionally exhibited amongst insects [8]. In terms of microscale swimming, the rheotaxis of squirmers, typically spherical particles that generate a fluid velocity at their surface, has been considered in detail [9,10], a biological example being the ciliated microorganism *Opalina* [11–13]. Squirmers, along with all microswimmers, may be classified hydrodynamically as either *pushers* or *pullers* depending on their method of locomotion [14]. A puller is characterized by drawing in fluid along one axis, before then expelling the fluid out at the sides, whilst pushers perform the reverse action, thus achieving propulsion in the opposite direction. In general the behaviors of the flagellated pushers are better studied than those of pullers, with particular recent focus on the boundary accumulation of flagellates such as the mammalian spermatozoon and the bacterium *E. coli* [2,15–18]. In particular, rheotaxis of mammalian spermatozoa has been known to occur for over 100 years [19], and has been recently explained in more detail with reference to its potential biological significance [1,6,20–22]. Given the lack of analogous knowledge regarding pullers, examples of which include the insect pathogens *Crithidia* spp. and related genera, as well as the genus of plant pathogens *Phytomonas*, there is therefore extensive scope to expand on current understanding and examine bulk puller response to background flows, as well as the additional effects of boundary proximity.

A further example of a hydrodynamic puller, the parasitic monoflagellates of the genus *Leishmania* are responsible for a major tropical human disease, leishmaniasis, which affects around 4 million individuals across the globe, from the Americas to the African continent [23]. Motile forms of *Leishmania* spp. utilize tip-to-base flagellar beating to achieve locomotion in the sandfly vector midgut, a feature of motile life cycle stages of all species of the family *Trypanosomatidae* including the aforementioned genera *Crithidia* and *Phytomonas* [24–29]. During their life cycle stage in the sandfly midgut, *Leishmania* promastigotes, characterized by their particular morphology, with a large cell body compared to their flagellum, are known to migrate toward the foregut following detachment from midgut epithelium [30]. They have also been observed at this stage to induce regurgitation in the vector by the secretion of promastigote secretory gel into their environment [31,32]. The precise role of such regurgitation is unknown, but is suggested by Walker *et al.* [33] to aid in the taxis of promastigotes in the bulk fluid. However, any possible rheotactic effects of background flows near boundaries have previously not been considered for *Trypanosomatidae*, even for Newtonian media, in contrast to their dynamics in quiescent fluid [33]. Thus our objective is to examine the effects of ambient Newtonian flows on the boundary swimming of this monoflagellated puller. Such a study will not only elucidate the behavior of *Leishmania* in experimental settings and microfluidic devices, but also inform our understanding of its behavior *in situ*, subject to the caveat that the rheology in the sandfly midgut is unknown.

To proceed we will investigate the response of motile monoflagellates to a shearing flow, with particular reference to the morphology and swimming characteristics of the *Leishmania mexicana* promastigote. Our candidate background flow, a shear flow, is one in which the fluid moves in nonmixing layers, may be generated experimentally near boundaries in microfluidic channels [4], and has previously been used to study the rheotaxis of human spermatozoa [1,2,6]. Indeed, the phenomenological model of Kantsler *et al.* [1] proposes a simple linear relationship between flagellate velocities and the parameters describing a background shear flow. If valid, such a model may be extended to the study of microswimmer populations, supplementing the prior descriptions of swimmer suspensions of Bearon and Grünbaum [34], Pedley and Kessler [35], and more generally facilitating further study into the behaviors of flagellate and active particle populations, with the latter as reviewed by Bechinger *et al.* [36]. Thus we additionally aim to ascertain to high precision the level of agreement between free-space flagellate swimming and a simplified description of their response to shear flows.

To proceed, we firstly recapitulate the boundary element formulation of the governing equations and detail the construction of a virtual swimmer. Further, we use the technique of phase averaging to provide an approximate quantification of virtual monoflagellate swimmers in free space and exposed to a shearing flow, drawing comparisons with classical studies of nonmotile bodies. We vary body lengthscales and flagellar kinematics in order to compare hydrodynamic classifications and the impacts of morphology in the scenarios of bulk and boundary swimming, and comment on the effects and the implications for guidance via time-dependent shearing rates. Our final objective is then to use the above to comment on the effects of shear flows on the boundary swimming of virtual *Leishmania*, and discuss potential relevance to *in vivo* promastigotes.

## II Methods

### A The virtual monoflagellate

**Fig. 1:**
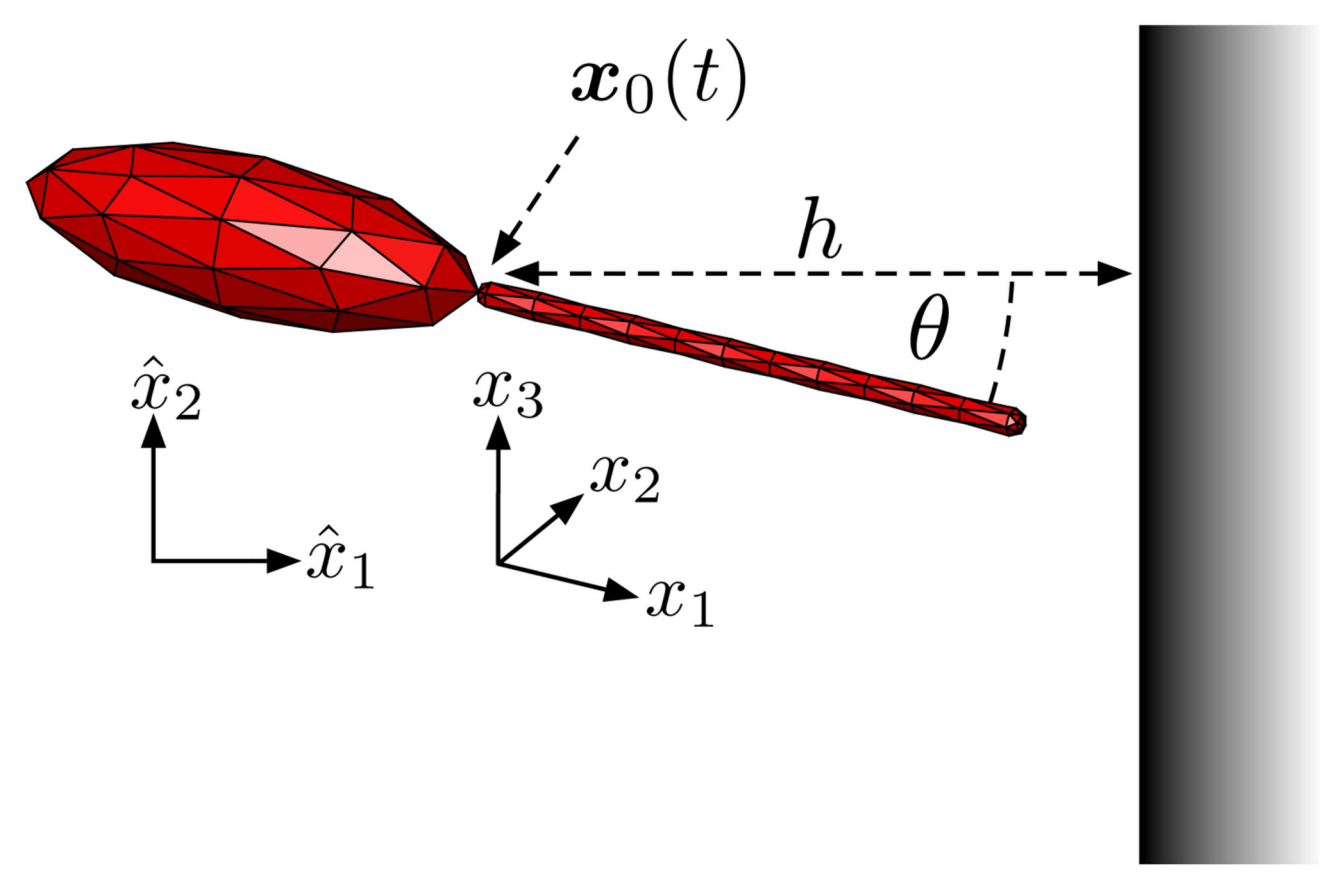
Computational representation of a monoflagellate. We model monoflagellated swimmers with an axisymmetric ellipsoidal body and an attached flagellum, as shown here for a virtual promastigote. The attachment location is denoted ***x***_0_(*t*) in the laboratory reference frame ${\hat{x}}_{1}{\hat{x}}_{2}{\hat{x}}_{3},$ and forms the origin of the swimmer-fixed reference frame *x*_1_*x*_2_*x*_3_. Planar boundaries will typically be specified by ${\hat{x}}_{1}=\text{const},$ with accompanying swimmer separation *h* and orientation *θ* shown.

In order to simulate the motion of monoflagellates, we utilize a general idealized computational representation: the virtual monoflagellate. Equipped with an axisymmetric ellipsoidal body and a long attached flagellum, the construction of a universal virtual monoflagellate enables the study of a variety of flagellated swimmers, and of perhaps most significance the *L. mexicana* promastigote. We model such a virtual promastigote as having a large prolate body, with typical major and minor axes of 11 and 3.5 *μ*m, respectively, and a flagellum of length 13 *μ*m, using the typical measurements of Wheeler *et al.* [37]. We define two reference frames, a laboratory and a swimmer-fixed frame, with coordinates ${\hat{x}}_{1}{\hat{x}}_{2}{\hat{x}}_{3},$ and *x*_1_*x*_2_*x*_3_, respectively, as shown in Fig. 1 and where the major axis of the ellipsoidal body lies along the *x*_1_ axis. The origin of the swimmer-fixed frame in the laboratory frame is denoted ***x***_0_(*t*), and is the location of flagellar attachment to the swimmer body. The surface of the monoflagellate is typically meshed using 644 triangular elements, with more elements being utilized for verification purposes and a coarse example being shown in Fig. 1.

To complete a kinematic description of the swimmer, we prescribe a planar beating pattern for the flagellum, as is evidenced to be typical for *Leishmania* in Fig. 1 of Walker *et al.* [33] and observed in other microswimmers, and adopt the convention that the beat plane lies on *x*_3_ = 0. In particular, we use the description of *Leishmania* beating identified by Walker *et al.* [33], with the flagellum centerline being parametrized explicitly by *ξ* in the swimmer-fixed frame as

(1) $$\begin{array}{l} x_{1}\left(\xi ,t\right)=\xi ,\\ x_{2}\left(\xi ,t\right)=A\left[\text{sin}\left(\frac{2\pi }{\lambda }\xi +2\pi ft\right)-\text{sin}\left(2\pi ft\right)\right],\\ x_{3}\left(\xi ,t\right)=0 \end{array}$$

for parameters *λ* = 13 *μ*m, *f* = 28 Hz, and *A* = 1.8 *μ*m. Here *ξ* ∈ [0, ξ*], where ξ* is chosen to conserve the flagellar length over time, and the form of *x*_2_(*ξ*, *t*) is such that the proximal base of the flagellum is attached to the swimmer body for all time *t*. The above parameter choices give a typical free-space swimming speed of approximately 1.5–2 *μ*m s^−1^, of similar magnitude to reported *Leishmania* motion *in vitro* [37]. We here note that the analysis that follows is robust to small changes in beating parameters, but that the full effects of significant deviation are not explored here in detail. When briefly considering the motion of pusher swimmers we will typically retain the virtual promastigote swimmer parameters, subject to reversals in beating direction and variations in body lengthscale, but no other morphological changes.

### B Governing equations and solution

The microscale dynamics of swimmers in a Newtonian medium are governed by the three-dimensional incompressible Stokes equations, which give the fluid velocity ***u***, expressed in the inertial laboratory frame, and pressure *p* as the solutions of the dimensional equations

(2) $$\mu \nabla^{2}\text{u}=\nabla p,\;\nabla \cdot \text{u}=0,$$

where *μ* is the dynamic viscosity of the fluid and these equations are imposed in the exterior of the domain Ω, which we will take to represent the exterior of the closed volume of a microswimmer. We enforce the additional conditions of force and torque-free swimming to close the system, as is typical for swimmers at small scale under the additional assumption of neutral buoyancy [14], removing the pressure gauge freedom as in Walker *et al.* [33]. The solution of these equations via the boundary element method is given by Pozrikidis [38], explicitly as the solution of

(3) $$\begin{array}{l} u_{j}\left({\text{x}}^{*}\right)=-\frac{1}{4\pi \mu }\int_{S}G_{ij}\left(\text{x},{\text{x}}^{*}\right)f_{i}\left(\text{x}\right)dS\left(\text{x}\right)\\ \;+\frac{1}{4\pi }\int_{S}^{PV}u_{i}\left(\text{x}\right)T_{ijk}\left(\text{x},{\text{x}}^{*}\right)n_{k}\left(\text{x}\right)dS\left(\text{x}\right), \end{array}$$

shown here for a surface *S* that will typically represent the surface of the swimmer. Here x* is a point on *S* with coordinates given in the swimmer-fixed frame, *f_i_* are the components of surface traction, and ***n*** is the surface normal directed into the fluid domain. Additionally, *G_ij_* and *T_ijk_* are velocity and stress Green’s functions of three-dimensional Stokes flow, and *∫^PV^* in the second summand denotes a principal value integral.

Prior to solution it is useful to decompose the fluid velocity into a known background and an unknown disturbance flow, denoted by ***u**_b_* and ***u**_d_*, respectively, expressed in the laboratory frame and following the work of Ishimoto and Gaffney [39]. Hence we prescribe ***u*** → ***u**_b_* in the far field and away from any boundaries, and the decomposition gives the appropriate condition at infinity for the unknown disturbance flow as ***u**_d_* → 0. On boundaries, including the swimmer surface, we impose the no-slip condition. In order to prescribe these conditions we may utilize the free-space or Blakelet integral kernels in Eq. (3) [40], with the latter additionally enforcing the no-slip condition on a specified planar boundary and noting that both choices yield solutions satisfying the far-field condition.

We may readily compute the solution of Eq. (3) for a known swimmer configuration in order to determine the instantaneous swimmer linear and angular velocities in the laboratory frame, taking *μ* throughout to be the dynamic viscosity of water at 25° C. We then employ a second-order time-stepping scheme to compute swimming trajectories, as detailed in Smith *et al.* [17].

The implementation of the boundary element method was verified against the work of Ishimoto and Gaffney [39], with sample simulations in agreement for the cases of bulk swimming and motion near a boundary. The integral kernels used in the solution of the boundary integral equations were compared with the implementations of Pozrikidis [38], showing agreement to machine precision. Final verification was also performed against the analytic solution of Jeffery [41] for passive ellipsoidal particles, with discretization parameters being chosen to give solutions of sufficient accuracy and precision. Where simulations include solid boundaries and will result in collision between the swimmer and the surface, simulations are halted when the distance between swimmer and boundary reaches ~2 nm.

**Fig. 2:**
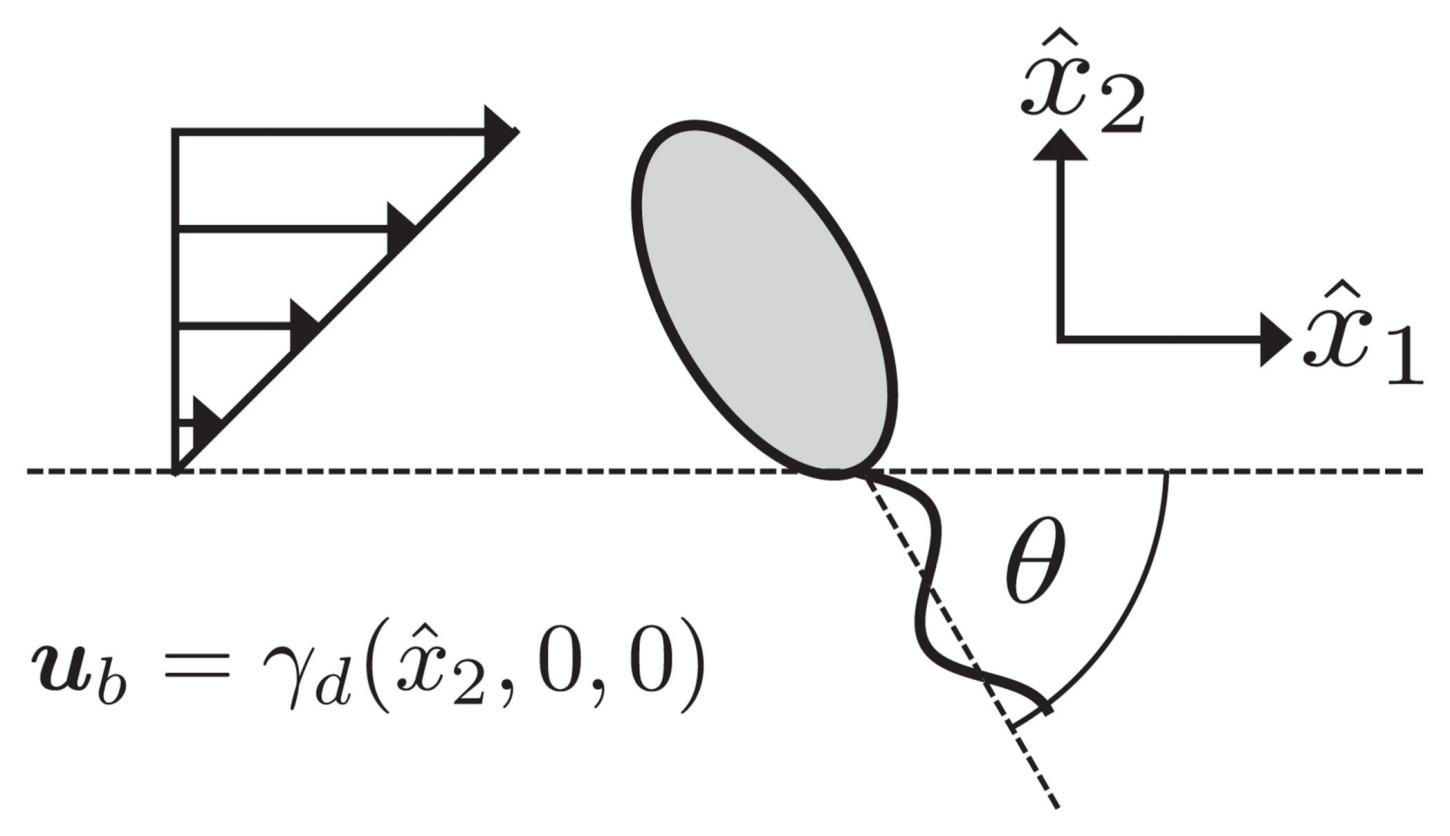
Describing planar flagellate motion in the bulk. Shown for a virtual promastigote, we define *θ* to be the clockwise angle between the swimmer-fixed major axis, *x*_1_, and the principal axis of the shear flow, ${\hat{x}}_{1},$ with the flow specified in dimensional form as ${\text{u}}_{b}=\gamma_{d}\left({\hat{x}}_{2},0,0\right),$ expressed in and relative to the laboratory frame. The monoflagellate’s beating plane lies in the plane of the background shearing flow (see Appendix for results concerning the more general case).

In studying the response of virtual monoflagellates to a shearing flow in the bulk we will prescribe ${\text{u}}_{b}=\gamma_{d}\left({\hat{x}}_{2},0,0\right),$ where *γ_d_* is a dimensional shearing rate and the frame coordinates are as shown in Fig. 2, whereas study near a planar boundary given by ${\hat{x}}_{1}=0$ will entail ${\text{u}}_{b}=\gamma_{d}\left(0,-{\hat{x}}_{1},0\right)$ to satisfy the no-slip condition at the boundary. A biologically appropriate choice of *γ_d_* is unknown, thus we will typically take *γ_d_* such as to give a nonwashout flow that nonetheless influences the swimmer.

### C Phase-averaged analysis

When investigating the long-time swimming of virtual monoflagellates we will often consider a phase-averaged analysis, utilized partly to reduce the computational costs of simulation, which can be prohibitively high due to the high mesh resolutions required for sufficient accuracy [33], but primarily to enable the application of dynamical systems theory in the study of swimmer motion. In order to obtain these averaged dynamics we simulate a swimmer in a given configuration at multiple points over its beat period, typically 20 or 40 sample points, and average the computed linear and angular velocities over the beat.

Additionally, in free space we will align the swimmer along the direction of shear, restricting the three-dimensional angular motion of the virtual flagellate to the beating plane. The orientation may then be described by the single clockwise angle *θ* as shown in Fig. 2. This significant restriction greatly simplifies the dynamics and enables analysis of an otherwise computationally complex scenario, exploiting body symmetry and the shearing nature of the background flow.

We make a similar simplification to the dynamics near a planar wall given by ${\hat{x}}_{1}=0,$ restricting the motion to a plane ${\hat{x}}_{3}=\text{const}$ and parametrizing by orientation *θ* and separation *h*. Here *θ* is defined as the clockwise angle between the swimmer-fixed *x*_1_ axis and the ${\hat{x}}_{1}$ axis of the laboratory frame, and the dimensional separation *h* is the perpendicular distance from the attachment point ***x***_0_(*t*) to the boundary, shown in Fig. 1. This gives a two-dimensional autonomous system, comparable with known analytic results for squirmers [42,43], and reduced from the four-dimensional dynamics that would allow rotations out of the plane of the shear.

The relevance of the study of these restricted dynamics in describing the full system is examined and evidenced in the Appendix. Comparisons between the predictions of the constrained dynamics and long-time simulations of the full dynamics, in particular pertaining to swimmers that are not aligned with or restricted to a plane, highlight remarkable agreement between the two systems, and thus justify the detailed study of the above dimensionally reduced systems in describing the behaviors of unrestricted swimmers.

### D Repulsive boundary forces

Reported *in vitro* for the bacterium *Staphylococcus aureus* by Klein *et al.* [44], strong repulsive boundary forces act between cells and boundaries. In order to capture the effects of such surface forces, including the effects of contact with the boundary, we can include short-range repulsive boundary forces of steric origin, given per unit surface area and scaling in strength with the ratio of fluid viscosity and dimensional beating period, *μ*/*T_d_*. Following Ishimoto and Gaffney [45], we explicitly give the force per unit area in dimensional form as

(4) $$f_{\text{wall}}\left(s\right)=g\frac{\mu }{T_{d}}\frac{e^{-s/l}}{1-e^{-s/l}}\text{n},$$

for outward-pointing unit boundary normal ***n***, boundary separation *s*, nondimensional scaling *g* = 1250 chosen to represent a strong force relative to other scales in the model, and characteristic decay length, *l*, that is much smaller than the cell scale, with *l* = 0.2 *μ*m. We will refer to a boundary equipped with this force as repulsive, whilst a boundary without such a potential will be described as passive.

## III Results

### A Virtual monoflagellate rotation in the plane may be partially approximated by passive ellipsoids in the bulk

**Fig. 3:**
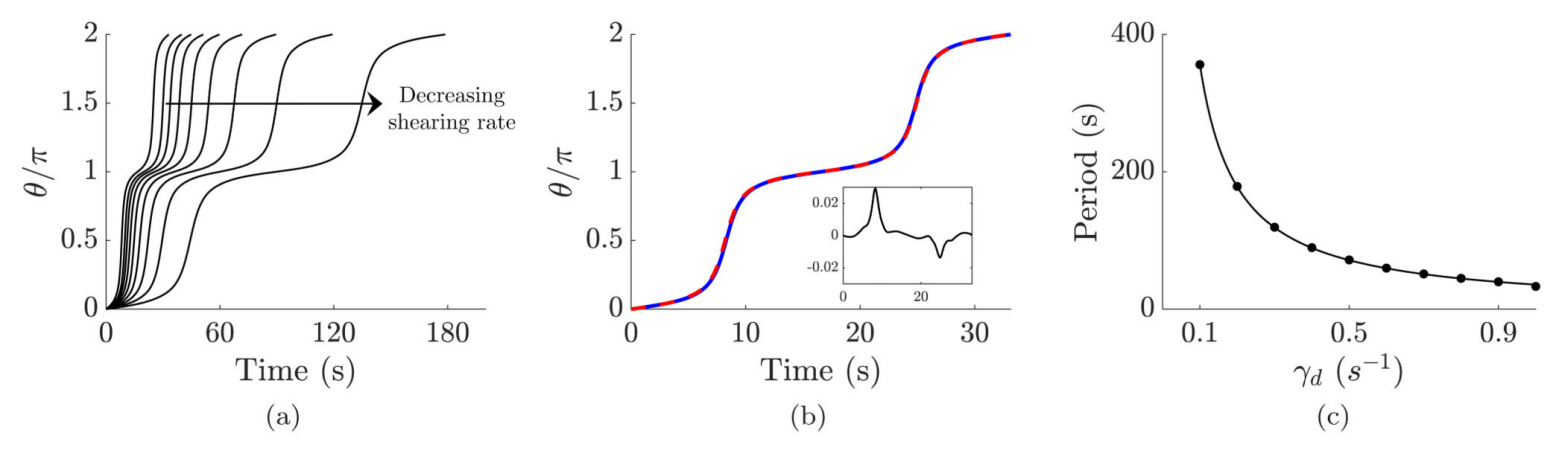
Free-space angular evolution of virtual monoflagellates, as predicted by phase averaging, in comparison to passive ellipsoids. (a) Temporal evolution of the angular displacement of a virtual promastigote in the bulk over one period, for background shear flow of shearing rates between 0.2 and 1 s^−1^. Here the period of rotation is seen to increase as shearing rate decreases. (b) The Jeffery’s orbit of an ellipsoidal particle in shear flow with the same period as the virtual promastigote is shown for *γ_d_* = 1 s^−1^ (blue, solid), showing remarkably similar dynamics to the corresponding promastigote orbit (red, dashed). The residual plot is inset, displaying error on the order of 10^−2^. (c) The dimensional period of promastigote rotation as a function of dimensional shearing rate (black dots). The periods of the promastigote rotation exhibit the same dependence on shearing rate as Jeffery’s orbits, as given by the smooth curve. Hence the interaction between the timescales of the background flow and flagellar beating are seemingly of little consequence in the phase-averaged system.

Due to a lack of symmetry-breaking features, in the bulk virtual pushers equipped with a planar flagellar beat are observed to turn in a shear flow aligned with their beat plane (see Fig. 2), rotating in unison with being guided by the background flow. The evolution and period of the rotation have been calculated using phase-averaged analysis, with the evolution of the angular displacement of the cell shown over dimensional time in Fig. 3(a). The phase-averaged planar motion is compared and verified against long-time simulation of the full system, and good agreement is observed. The angular motion is similar in character to the well-known Jeffery’s orbits of passive ellipsoidal particles in shear flow [41]. Thus the orbits were compared against the dynamics of axisymmetric ellipsoidal particles with appropriate dimensional period of rotation *T_d_*, choosing the aspect ratio *r* > 1 of each ellipsoid as that given by Jeffery’s formula,

(5) $$T_{d}=\frac{2\pi }{\gamma_{d}}\left(r+\frac{1}{r}\right).$$

One such comparison is shown in Fig. 3(b), between a passive ellipsoid and an active virtual promastigote, where only slight quantitative differences are observed between the two. Thus the phase-averaged angular dynamics of our large-bodied puller may be approximately captured by a Jeffery’s orbit for a given shear rate, but the same ellipsoid *a priori* may not be a suitable replacement across a range of shearing rates. In fact, analysis of the dimensional period *T_d_* as a function of shearing rate *γ_d_* revealed that a single choice of ellipsoid is indeed appropriate for a range of background flow rates (*r* ≈ 5.49 for the virtual promastigote), and shows remarkable agreement with the functional form given in Eq. (5), as shown in Fig. 3(c). Hence we conclude that the rotational motion of flagellated pullers with large cell bodies and a planar beating pattern may be reasonably approximated by the Jeffery’s orbit of an appropriate passive ellipsoid, the aspect ratio of which may be determined by simple phase plane analysis and curve fitting.

By the time reversibility of the governing equations and boundary conditions, this method of approximation is also valid for a pusher with the same morphology and beat characteristics, obtained as a result of reversing the direction of flagellar beating. Thus the efficacy of approximating angular dynamics with Jeffery’s orbits is independent of whether the swimmer is a pusher or a puller.

**Fig. 4:**
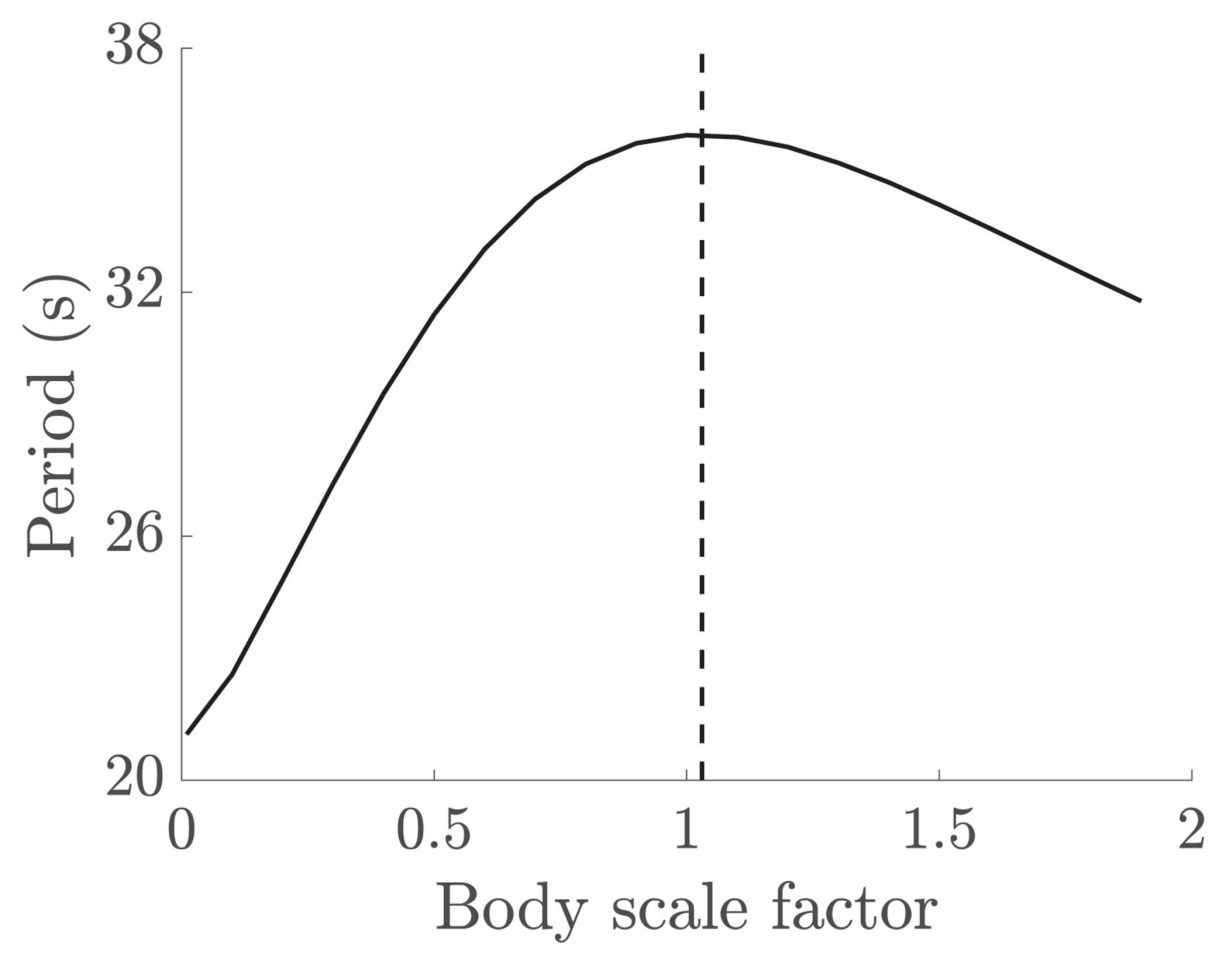
Period of rotation for virtual puller monoflagellates in shear flow of rate *γ_d_* = 1 s^−1^, for a range of body lengthscales. Body lengthscale is shown as a scaling factor applied to the typical pro-mastigote body parameters introduced in Sec. II A, where a scaling of 1 corresponds to the typical *Leishmania mexicana* lengthscale. The maximum period of rotation is highlighted by the dashed vertical line and occurs for a scaling ~1, which corresponds approximately to a typical promastigote.

The above analysis was repeated for a range of body lengthscales, whilst retaining body symmetry and flagellum lengthscale, with the resulting periods of rotation shown in Fig. 4. Here we have fixed the shearing rate *γ_d_* = 1 s^−1^, along with flagellum morphological and beating parameters. The resulting curve highlights a maximal period of rotation, corresponding approximately to the body lengthscale of the virtual promastigote. Thus *L. mexicana* promastigotes of typical length appear to exhibit the largest period of rotation for variations in body scale.

### B Guided bulk swimming may be achieved using temporally evolving shearing flows, and readily approximated

Coupling the angular evolution of a monoflagellate with its linear velocity completely describes the phase-averaged planar motion of such a swimmer. Computing the phase-averaged linear velocity of the swimmer for given background flow rate *γ_d_* and orientation *θ*, notably less expensive computationally than full long-time simulation, it is possible to simulate the approximate long-time motion of the microswimmer and determine the effects of a variable background shearing rate. It is thus feasible to obtain an approximate swimming trajectory without large computational cost given a prescribed time-varying background flow.

**Fig. 5:**
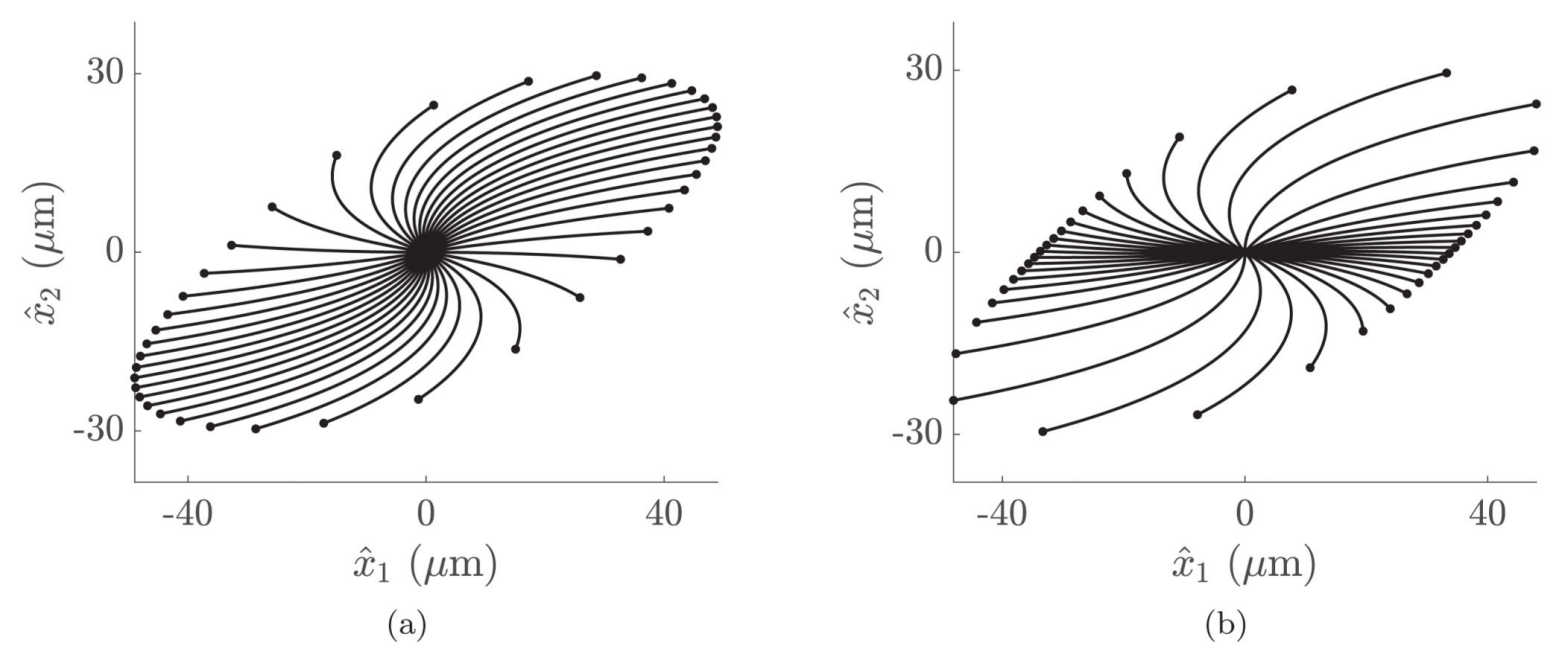
Swimmer trajectories computed from phase-averaged analysis, initialized at the origin of the laboratory frame in a background shear flow and shown after 2 s. (a) Trajectories with initial orientation sampled from a uniform distribution. The density of endpoints provides a naive estimate of the probability of initially randomly oriented swimmers being in a given region after a specified time. (b) Trajectories where initial orientation is sampled according to the distribution of orientations during a Jeffery’s orbit. A differing distribution of endpoints to (a) can be clearly seen, giving a markedly different but physically realistic quantification of how the location evolves for a swimmer with unknown orientation. Here the background shear flow is prescribed in the laboratory frame as ${\text{u}}_{b}=\gamma_{d}({\hat{x}}_{2},0,0),$ where results are shown for *γ_d_* = 1 s^−1^ and are qualitatively robust to changes in both shear rate and simulation length.

Via the same method it is also possible to approximately determine the set of all possible swimming paths from a given configuration for a specified background flow. We consider a swimmer in a background shear flow ${\text{u}}_{b}=\gamma_{d}\left({\hat{x}}_{2},0,0\right),$ expressed in the laboratory frame and here with *γ_d_* = 1 s^−1^. Additionally assuming that the initial swimmer position is known, without loss of generality we take this position to be the origin of the laboratory frame. Supposing that the unknown initial orientation of the swimmer is distributed uniformly in [0, 2*π*], as might be a naive assumption for an unknown flagellate, we see in Fig. 5(a) that the most probable positions after some fixed time may be identified with the regions of increased point density, with paths being shown after 2 s and qualitatively robust to large variations in simulation interval and shearing rate. Refining the distribution of initial orientation using the dynamics of the Jeffery’s orbit approximation, we obtain the profile shown in Fig. 5(b), exhibiting a stark contrast in distribution and showing a greater likelihood of travel along the axis of the flow than the naively distributed approach, as might be reasonably expected.

**Fig. 6:**
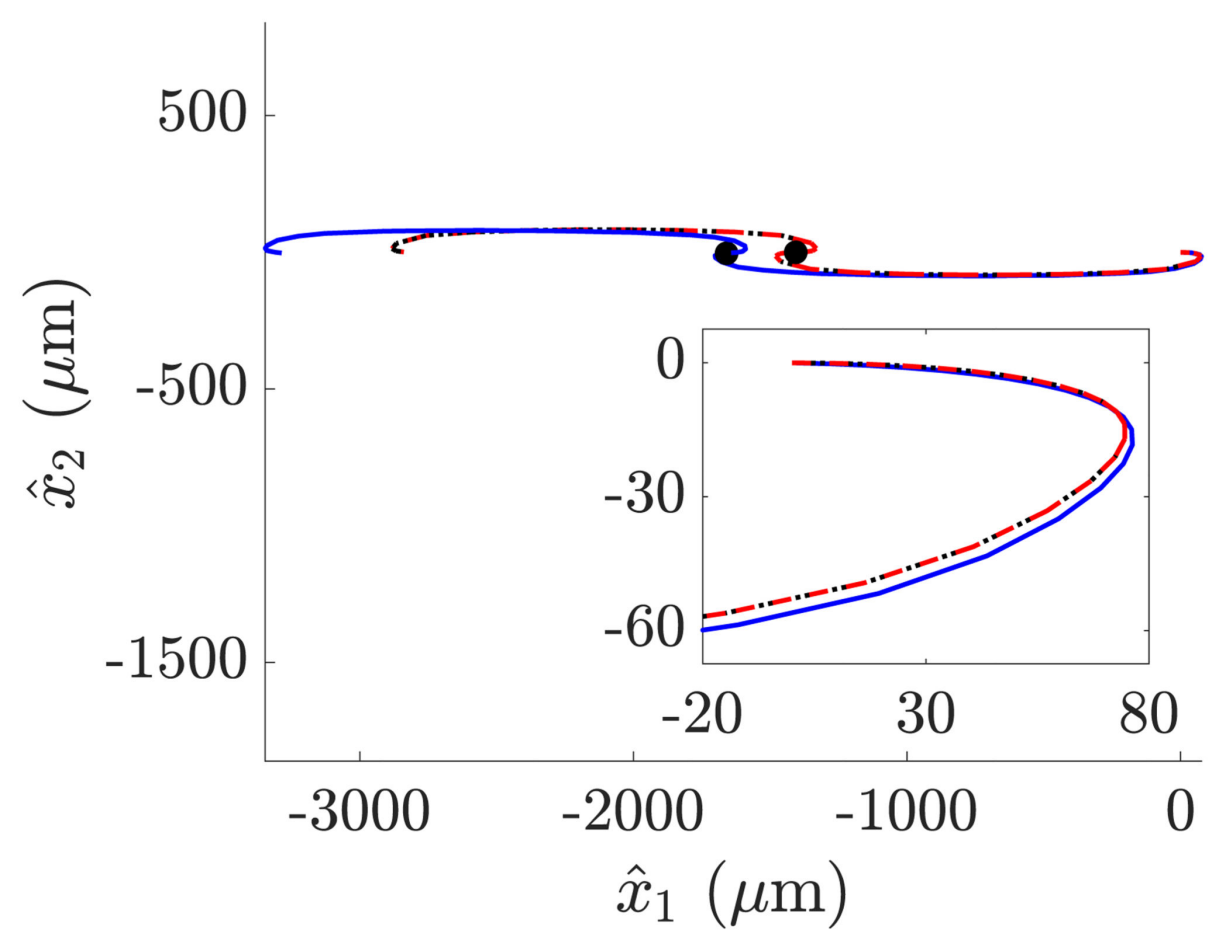
The shear-guided swimming path of a monoflagellate in a specified time-dependent shear. The phase-averaged approximation (red, dashed) is observed to closely resemble the long-time simulation of the swimmer (blue, solid), beginning from the origin of the laboratory frame with *x*_1_ parallel to ${\hat{x}}_{1}.$ The locations of background flow reversal, corresponding to a specific time and switching the shearing rate from *γ_d_* = 1 s^−1^ to *γ_d_* = −1 s^−1^, are highlighted on the paths (black, filled). The prescribed path is shown as a black dotted curve, seen to coincide with the phase-averaged approximation. Inset is the initial section of the trajectory. Quantitative agreement is improved with increased refinement of the phase-averaged and long-time simulations, but computation of the latter is prohibitively expensive. Overall good agreement over such a timescale (60 s) validates the phase-averaged approximation and the resulting method of path prediction.

In addition, we theorize that one may inform the specification of background flows using the same phase-averaged analysis in an attempt to prescribe a swimming path, thereby enabling the guidance of a swimmer along a specified path by dynamic changes in background shearing rate. As an example of this, a simple path shown as a black dotted curve in Fig. 6 was prescribed for the typical virtual promastigote, informed by Fig. 5 and aiming to guide the swimmer in a fixed direction. Taking the intended direction of guidance to be along that of the shear flow without loss of generality, an appropriate shearing rate evolution was determined from phase-averaged analysis. For such a rotationally symmetric path only a single shear reversal is required, with the reversal time being half the period of rotation. Long-time simulation of the swimmer in this time-dependent background flow illustrates good agreement between the predicted and fully simulated paths, particularly on a short timescale. Agreement is improved by refining the simulation of the full system, and thus the comparison validates the efficacy of both the phase-averaged approximation and the proposed method of swimmer guidance.

### C Shearing flow in general does not induce stable boundary taxis in virtual promastigotes

**Fig. 7:**
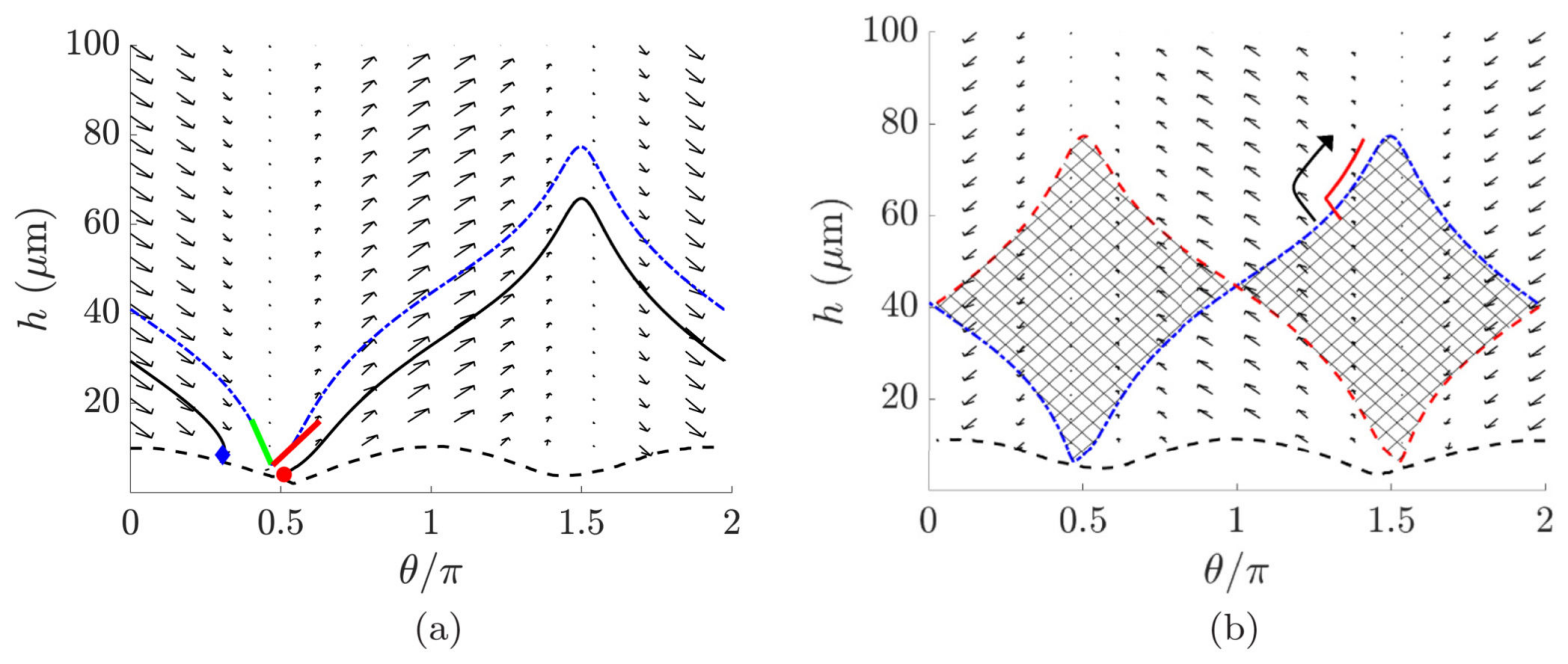
Phase planes describing the phase-averaged dynamics of a large-bodied puller in shearing flow of rate *γ_d_* = 1 s^−1^ near a no-slip planar boundary. The black dashed line separates off the region of the phase plane where configurations intersect with the boundary. (a) Approximate stable and unstable manifolds of the saddle in 0 < *θ* < *π*/2 are shown as green (left) and red (right) curves, respectively. A sample trajectory exemplifying boundary collision is shown as a black curve, with the start and endpoints shown as a red circle and a blue diamond, respectively. The separatrix, partitioning the phase plane into collision or bulk-bound configurations, is shown as a dot-dashed blue curve. (b) The phase plane corresponding to a reversal of shear direction from (a). The separatrix corresponding to the original flow direction (blue, dot-dashed) is shown superimposed upon the phase plane, a mirror of the reversed separatrix (red, dashed). The cross-hatched region lying between the two curves includes those configurations whose characteristic long-time behavior will be changed by a shear reversal. The smoothed path of a sample long-time simulation in the reversed flow is shown (red, solid), which crosses the original separatrix from beneath. Following the crossing, if the flow direction is reversed, the dynamics become those represented in (a), changing the long-time behavior of the swimmer, with the trajectory following the direction of the large black arrow.

In order to characterize behaviors of virtual monoflagellates near a passive no-slip boundary in the presence of shear flow, we compute a time-averaged phase plane as described in Sec. II C, where orientation *θ* is as shown in Fig. 1. An example of such a phase plane is shown in Fig. 7(a), where we have simulated a virtual promastigote in moderately strong shear flow of dimensional shearing rate *γ_d_* = 1 s^−1^ parallel to the boundary, with the flow direction being such that $\dot{\theta }>0$ in the absence of boundary effects. From the phase plane we identify a fixed point of the system in 0 < *θ* < *π*/2, corresponding to the balance of torques from the rotational background flow and attractive hydrodynamic boundary effects. The fixed point is seen to be a saddle, and thus unstable, with approximate stable and unstable manifolds being shown in Fig. 7(a). Further, noting the periodicity of the system in *θ*, we observe a homoclinic orbit connecting the saddle to itself, creating a separatrix between the behaviors of boundary trapping and deflection into the bulk.

Here, with a passive no-slip solid boundary, we observe that configurations on one side of the separatrix will eventually result in boundary collision, with the lower branch of the unstable manifold preventing periodic swimming and thus lasting accumulation. Conversely, on the other side, boundary escape is predicted. These behaviors were confirmed by long-time simulation of the full system, and observed to be robust to perturbations in body lengthscale and flagellum beating parameters, as well as significant changes in background flow rates, where *γ_d_* ranges between 0.01 and 2 s^−1^.

A reduction in shearing rate sees the separatrix retained, but situated at increased values of separation *h*, with the saddle moved correspondingly. An increase in shearing rate *γ_d_* from the reference value of 1 s^−1^ to above a critical value, computed for our virtual promastigote to be *γ_d_* ≈ 1.2 s^−1^, results in the stationary point being in such close proximity to the boundary that configurations beneath the separatrix effectively represent immediate boundary collision. Thus in high shear we enter a parameter regime where the steady state approximately corresponds to collision with the surface, resultant of the large-magnitude torque exerted on the swimmer by the background flow. However, at such flow rates the details of microswimmer morphology and flagellar beating are subdominant to the effects of the background flow and washout, thus we will not consider high flow rates further. For all flow rates of physical interest we therefore conclude that the same behavioral dichotomy exists with no possibility of periodic swimming, and thus virtual promastigotes will not in general boundary accumulate in shear flow.

### D Boundary behaviors may be controlled via variable shearing rates

Recalling from Sec. III C that decreasing background flow shear reduces proximity of the separatrix to the wall, we note that toggling cell behavior between wall escape and attraction for a given location and orientation may be effected by altering shear. In order to change the swimming behavior an appropriate change in shear is required. From a sample phase plane [see Fig. 7(a)] we see that in fact an instantaneous change from a shearing rate *γ_d_* to the reverse flow (*γ_d_* ↦ −*γ_d_*) will achieve behavior reversal in a large proportion of reasonable cases, simply by noting that a reversal in shear direction is equivalent to the mapping *θ* ↦ 2*π* – *θ*, and thus gives a separatrix mirrored about *θ* = *π*. A sample trajectory illustrating this effect is presented in Fig. 7(b).

Whilst instantaneous changes in background flow are acceptable in the inertia-free limit of Stokes equations, it is not clear that such immediate changes in shear can be performed in practice. However, we observe that the same behavioral change may be obtained from a continuous variation in shearing rate, exploiting the response of the separatrix to a change in shearing rates, and, in particular, without the need for an explicit and instantaneous background flow reversal. Hence we conclude that the long-time boundary behaviors of a large-bodied puller may be dynamically adjusted via background flow calibration.

### E Repulsive boundary character combined with shear flow may give rise to boundary swimming in virtual promastigotes

Whilst we have observed that a shear flow alone is not sufficient to induce stable boundary swimming in a large-bodied puller like the virtual promastigote, we investigate a boundary of different character by introducing a short-range repulsive boundary potential, simulating a contact force or other strong repulsion close to the boundary, as discussed in Sec. II D. Due to the short range of the repulsion, it is not in general appropriate to use the method of phase averaging to study the near-boundary behavior of flagellates due to the flagellar beat, typically possessing an amplitude larger than the characteristic range of the boundary force and thus rendering phase averaging unreliable. However, in the medium to far field of the boundary the use of the phase-averaged dynamics is justified, as the surface potential is of negligible magnitude away from the boundary. Hence we use long-time simulation in conjunction with the phase planes of Sec. II C to investigate the effects of a repulsive boundary potential on monoflagellate swimming.

**Fig. 8:**
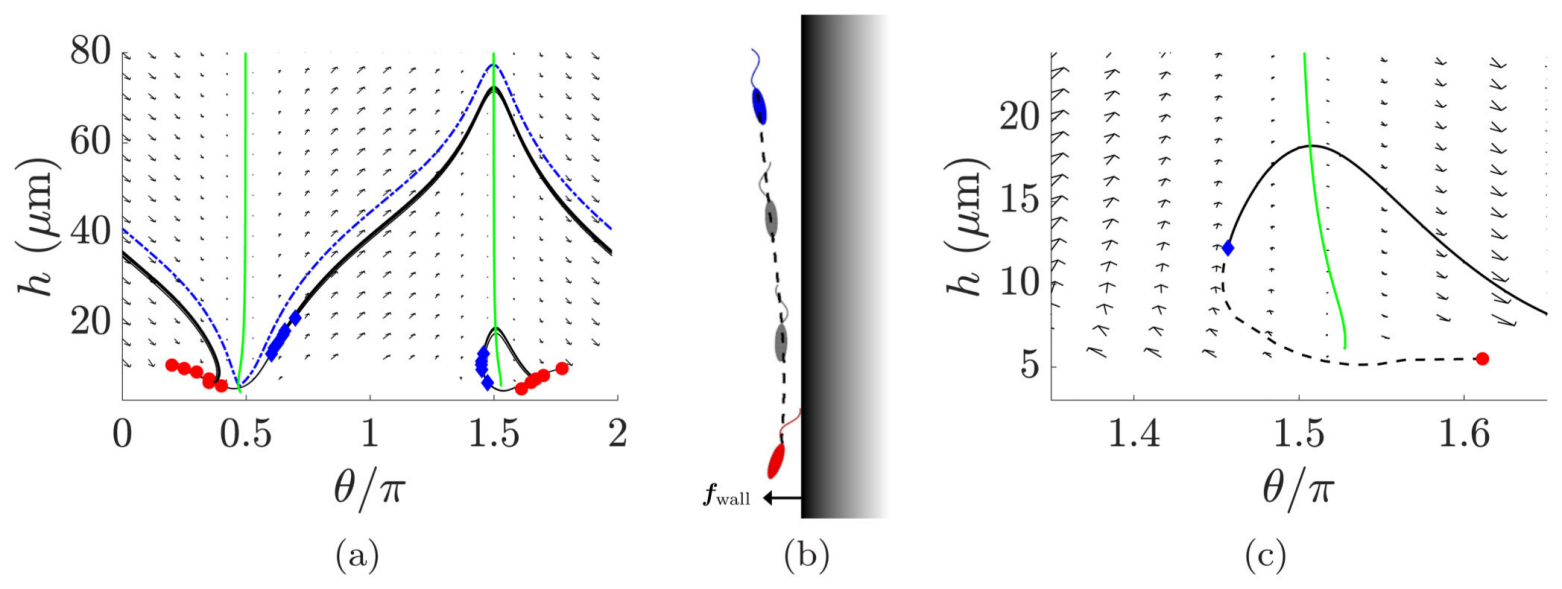
Motion of virtual promastigotes in a shear flow near a repulsive planar boundary. (a) With the *h* nullcline displayed in green (approximately vertical) and the separatrix displayed as a blue dot-dashed curve, multiple trajectories are shown across the phase plane, with start and endpoints of full simulations being shown as red circles and blue diamonds, respectively. Shown in black are smoothed traces of motion, resultant of simulating the full dynamics, in addition to their phase-averaged continuations, demonstrating a repeated washout tumbling for configurations beginning near the saddle point, and quasiperiodic behavior for those around *θ* = 3*π*/2. (b) Results of a full simulation of motion, with the first and last frames displayed in red (lower) and blue (upper), respectively. (c) A region of the phase plane representing the motion of (b), with the endpoints of the full simulation highlighted. The *h* nullcline is shown in green (approximately vertical), with the smoothed trajectory shown in black, dashed. At the end of the full simulation the repulsive boundary force becomes negligible, hence the dynamics then follow the phase plane on a collision-bound trajectory (black, solid).

Simulating the long-time motion of a virtual promastigote in close proximity to the boundary in significant shear flow, we observe a qualitative change in overall swimmer behavior. To see this we consider the example phase plane of Fig. 8, computed for shearing rate *γ_d_* = 1 s^−1^ for the promastigote-type swimmer, along with the full simulation of the near-boundary dynamics exemplified in Figs. 8(b) and 8(c). Beginning from any given initial configuration (examples shown in red in Fig. 8), we simulate the full system to accurately determine the motion induced by the boundary repulsion. As the flagellum approaches the boundary, promoted by the tip-first swimming of the flagellate, the strong boundary force exerts a torque on the swimmer, causing it to rotate away from the surface. Following this reorientation, the swimmer evolves to a configuration away from the surface in which the repulsive force is again subdominant, with the flagellum directed into the bulk. For swimmers initially near *θ* = 3*π*/2, we then note from the phase plane that the resulting configuration (shown in blue) is greatly beneath the separatrix, and thus the motion that follows will entail the swimmer again drawing close to the boundary (shown in black), where the process will repeat following an apparent limit cycle. For configurations that are initially close to the saddle point at *θ* ≈ *π*/2, as can be seen in Fig. 8(a), we observe a repeated tumbling motion, with the repulsive surface force allowing swimmers to cross the unstable manifold of the saddle in phase space and remain beneath the separatrix. The swimmer then moves off into the bulk, but is still captured by the shear flow and again draws close to the boundary. During this cycle the swimmer is largely far from the boundary and facing downstream (*θ* > *π*), and hence is convected predominantly with the background flow.

Thus we have identified two modes of downstream quasiperiodic boundary swimming of virtual promastigotes in the plane, one in which swimmers remain facing downstream and another in which the virtual flagellate tumbles. In the former case the rotational effects of the shearing flow cause the forward-facing flagellum of the puller to approach the boundary, where it is then subject to significant repulsive forces and subsequently reoriented back away from the boundary. This repeated behavior is not seen in pushers in the same way, exemplified by the well-known upstream stable boundary swimming of the human spermatozoon in a shear flow [1,6,20–22]. We do, however, retain the quasiperiodic swimming in pullers with reduced body lengthscales, having repeated the above analysis for a swimmer with body length-scale decreased by 80% from that of the virtual promastigote. This suggests that the presence of such a behavior is reliant on a hydrodynamic mechanism and independent of body lengthscale.

## IV Discussion and Conclusions

In this work we have explored the response of idealized virtual microswimmers to a shearing flow, examining the resulting behaviors of the flagellates and their dependence on swimmer type and morphological scale. We have compared the planar orbits of virtual monoflagellates aligned with shear flow to the Jeffery’s orbits of passive ellipsoidal particles, observing remarkable agreement and thus justifying the use of the latter as an approximation of the angular evolution of the former. Considering the resulting period of orbit as a function of the body lengthscale, we observed a scaling that gives rise to virtual promastigotes undergoing a maximal period of rotation, for typical parameters used in the study of *L. mexicana* promastigotes [33,37], suggesting some notion of optimality in *L. mexicana* morphology. However, due to morphological differences and natural variation in promastigotes within the cell cycle and across life cycle stages [37,46], there may be little biological significance of this property, if indeed the property holds for nonidealized swimmers *in vivo*.

Following the discovery of Jeffery’s orbits as an appropriate approximation to the angular motion of our monoflagellates, we then demonstrated that the linearity inherent in the orbits of these passive particles may be exploited to enable computationally feasible approximation of long-time trajectories of the planar motion of monoflagellates. This in turn may be utilized for flow-driven swimmer guidance in the bulk. Whilst variations in body lengthscales were considered, the effects of symmetry-breaking morphology were not examined in detail. Indeed, due to the rich character of Jeffery’s orbits for nonaxisymmetric particles, as reported classically by Hinch and Leal [47], we expect the effects of asymmetry to depend strongly on the particular morphology of the swimmer, and is for future consideration.

Nonetheless, for axisymmetric swimmers moving in a plane we have demonstrated that swimming paths may be predicted with significant accuracy and low computational cost, enabling further study of swimmer response to bulk guidance cues. Additionally, as explicitly demonstrated in the Appendix, such dynamics also tracks the projection onto phase space of trajectories that are not restricted to the plane of shear. Furthermore, the Jeffery’s orbit dynamics, coupled with progressive velocity, entails a verified description of the monoflagellate swimmers that is very simple, with further possibilities in exploring such approximations in more complex background flows, applied to other swimmers and population dynamics. Indeed, the phenomenological model of rheotaxis proposed by Kantsler *et al.* [1] is here justified physically (at least in projection for the out-of-plane dynamics) by our comparison of monoflagellate rotation with Jeffery’s orbits, verified by the phase-averaged path analysis of Sec. III B. We further propose that the two-dimensional models of Bearon and Grünbaum [34] and the continuum formulation of Pedley and Kessler [35] may be augmented with the analytic solution of Jeffery’s orbits in order to simulate the approximate collective motion of a population of flagellated swimmers.

We have additionally produced a qualitative description of the distribution of trajectories for a randomly oriented swimmer in a prescribed shearing flow, comparing naive and informed distributions of initial flagellate alignment. We observed a significant bias for motion along the direction of shear, as would be expected for passive particles, demonstrating further the similarity in behaviors between passive ellipsoids and motile flagellates of this type.

Further, we have investigated the impacts of a passive planar no-slip boundary on how shearing flows affect monoflagellates. In particular, we have observed a lack of stable boundary swimming in the planar behaviors of virtual *L. mexicana* promastigotes, additionally demonstrating that swimming stability is not qualitatively dependent on moderate changes in body lengthscale. Following from the pusher-puller duality of microscale swimming, which arises from the time reversibility of Stokes equations and accompanying boundary conditions, in this case we may identify the behavior of a puller as the time reversal of the morphologically equivalent pusher. From this we see that the lack of stable swimming behavior observed here in pullers is consistent with the recent analysis of monoflagellated pushers [17,18,39,48,49], which are seen to stably accumulate near boundaries, although the findings here may not be immediately inferred due to the large cell body of *Leishmania* compared to pusher monoflagellates such as sperm.

We have explored and classified the behaviors via the use of a separatrix, a feature of the phase-averaged planar dynamics that partitions the swimmer behavior based on its instantaneous configuration. Analysis of the dependence on shearing rate of this curve in two-dimensional configuration space suggested a method for controlling a swimmer near boundaries, providing a quantitative description of the effects of time-varying background flows on long-time swimming behavior. Indeed, we have demonstrated that flow reversal may be capable of switching swimmer behavior between boundary escape and attraction *in vitro*, and have further shown that similar results may be obtained by continuous changes to background flow rates.

Changing the character of the planar boundary from a passive to a repulsive surface, we have noted the exhibition of boundary swimming in the behaviors of idealized pullers exposed to shear flow. Identifying this virtual swimmer with the *L. mexicana* promastigote suggests a potential accumulation behavior of *in vivo* parasites in any rotation-inducing background flow, although the effects of symmetry-breaking morphological differences between the two swimmers and non-Newtonian media remain to be investigated in detail. Nevertheless, following the direction of the background flow this downstream shear-resultant taxis is markedly different from the documented upstream rheotaxis of flagellated pushers [2,17,18,39,48,49], and notably is exhibited across a range of body lengthscales.

As detailed in the Appendix, we have seen that the rich and complex dynamics of the two-dimensional subspace we have considered is highly representative of the full dynamics for behavior near boundaries, at least in projection onto the height, *h*, and the angle, *θ*, and thus not recording the orientation dynamics. Furthermore, the observed lack of dependence of the behaviors on the details of the swimmer body suggests that the nature of boundary accumulation in monoflagellates is more significantly dependent on the hydrodynamic classification of the swimmer, with the boundary swimming of pullers only quantitatively reliant on body lengthscales.

In summary, we have considered in detail the responses of virtual monoflagellated swimmers to a shearing background flow, exploring the effects of varying body lengthscales, shear strengths, and the hydrodynamic classification of the swimmer. We have seen that in the bulk we may reliably utilize the Jeffery’s orbits of passive particles to approximate the rotational dynamics of both hydrodynamic pushers and pullers, having demonstrated the efficacy of phase-averaged approximations of motion and a proposed method of bulk swimmer control. Having sought to determine the impact of a planar boundary on swimming, we observed an absence of boundary swimming in virtual promastigotes, in contrast to the widely reported behaviors of monoflagellated pushers. Further, the identification of a phase-averaged separatrix highlighted a dichotomy of behaviors, with configurations split between unimpeded motion in the bulk and collision-bound swimming. Introducing a repulsive surface potential, we saw the emergence of quasiperiodic downstream rheotactic boundary swimming in idealized promastigotes, driven by oscillatory switching in the dominance of boundary forces and shear-induced rotation. Finally, we have demonstrated a potentially general method for realizing individual swimmer guidance, in principle enabling the dynamic guidance of a variety of motile monoflagellates in microfluidic devices.

The research materials supporting this article have been made available [50].

## Supplementary Material

Appendix
