# Supplementary material for: Response of monoflagellate pullers to a shearing flow: A simulation study of microswimmer guidance
Source: Phys Rev E. Author manuscript; Available in PMC 2019 Jan 15. (PMC6333290)
Supplement: Appendix [file NIHMS80764-supplement-Appendix.pdf]

APPENDIX

We establish the relevance of the two-dimensional phase-averaged subspace described in Sec. II C to the full dynamical system representing monoflagellate motion near a boundary. To fully describe the three-dimensional orientation of a monoflagellate we define the Tait-Bryan angles ψ, ϕ, and θ. These are taken to represent successive rotations about the laboratory axes $\hat{x}_1$, $\hat{x}_2$, and $\hat{x}_3$, respectively, and with θ the analog to that defined in Figs. 1 and 2, where the inertial frame axes are oriented relative to the shear flow in the absence of a wall (Fig. 2) and relative to the wall in its presence (Fig. 1). In particular, the orientation of the swimmer before rotation is such that the axes of the laboratory and swimmer-fixed frames align, so that $\psi = \phi = \theta = 0$ describes a configuration with the swimmer beat plane parallel to the plane of the shear flow and perpendicular to the boundary. As in the main text we denote the separation of the origin of the swimmer-fixed frame from the wall by h.

In Fig. 9 we present three examples of unrestricted virtual promastigote motion in the presence of a repulsive or passive planar boundary, along with a shear flow as described in Sec. II B, in each case the initial orientation of the swimmer being taken to be a perturbation away from a planar configuration (see Fig. 9 for details). Comparison of the trajectory of Fig. 9(a) with the periodic downstream swimming of Fig. 8(a)

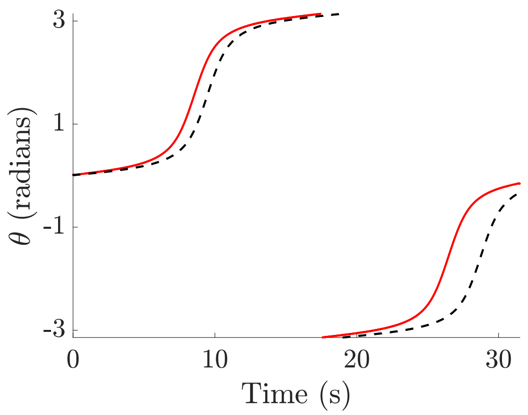

FIG. 10. Sample long-time simulations of the motion of a virtual promastigote in the bulk in a background shear flow. The smoothed evolution of the planar Tait-Bryan angle θ is shown for a swimmer oriented within the plane of the shear flow (red, solid) and for a swimmer with a nonplanar initial configuration (black, dashed), demonstrating good agreement over a long timescale. Initially $(\psi, \phi, \theta) = (-0.79, 0, 0)$ for the nonplanar swimmer, with all angles being given in radians. The discontinuity in the plot simply arises as the range of θ is $[-\pi, \pi)$.

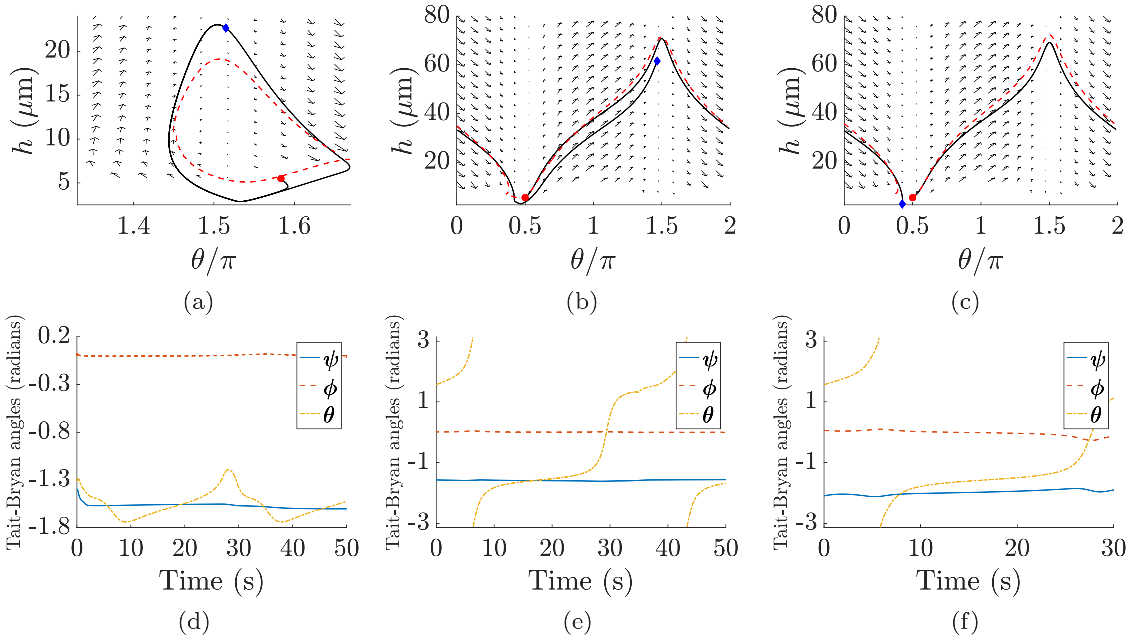

FIG. 9. Sample long-time simulations of the motion of a virtual promastigote near a planar wall in shear flow from various nonplanar initial configurations. (a)–(c) Projections of the motion onto the h-θ space, allowing direct comparison of trajectories with those of Figs. 7 and 8. The start and endpoints of the motion are shown as red circles and blue diamonds, respectively, with the projected, smoothed path displayed as a black curve and showing remarkable qualitative agreement with those presented in Figs. 7 and 8. Sample paths of the planar system are shown as red dashed curves for comparison. Here (a) and (b) correspond to the repulsive boundary of Fig. 8, whilst (c) is analogous to the passive boundary of Fig. 7. The trajectory shown in (c) is truncated at 30 s as the swimmer collides with the boundary. (d)–(f) The smoothed evolution of the swimmer Tait-Bryan angles corresponding to the trajectories of (a)–(c). Periodic nonplanar motion is exemplified in (d), whilst the angles corresponding to out-of-plane rotation in (e) and (f) can be seen to evolve in general on a slower timescale than the in-plane angle θ, in agreement with the general observation of Ishimoto [10]. Initial conditions are (a) $(\psi, \phi, \theta) = (-1.40, 0.02, -1.31)$, (b) $(\psi, \phi, \theta) = (-1.57, 0, 1.57)$, and (c) $(\psi, \phi, \theta) = (-2.10, -0.05, 1.58)$, with angles given in radians and $h = 5.5\ \mu$m in all cases. Discontinuities in (e) and (f) arise as the range of θ is $[-\pi, \pi)$.

demonstrates clear agreement between the behaviors of the full and restricted systems, with the projection of the full dynamics onto the h-θ space closely matching the computed trajectories of the two-dimensional subspace. The approximate periodicity and nonplanar nature of the motion in the full system is evident from the angular evolution shown in Figs. 9(a) and 9(d), where we observe an initial transient from a random initial state to an apparent periodic nonplanar motion, with multiple cycles of this repeating behavior being shown. This remarkably demonstrates the existence and stability of a downstream swimming behavior in the full dynamics, corresponding to the periodic motion reported in Sec. III E.

Similarly, in comparison with Fig. 8(a), the phase-plane projections and time series of Figs. 9(b) and 9(e) highlight the existence of quasiperiodic tumbling in virtual monoflagellates in the full dynamics, as found as a feature of the planar dynamics in Sec. III E. We observe little change in ψ or ϕ in comparison to the large variation in θ throughout the motion, supporting the conclusion of Ishimoto [10] that out-of-plane dynamics proceed on a slower timescale than the in-plane dynamics. The same level of agreement is also present in the case of a passive boundary, as in part demonstrated by Figs. 9(c) and 9(f), showing the projection of a nonplanar swimming path that ends in collision with the boundary, as predicted by the planar dynamics. Further, and in supplement to the agreement seen in the far field in Fig. 9, clear correspondence between the behavior of virtual swimmers in the bulk is exemplified in Fig. 10, where good agreement between the angular evolution of planar and nonplanar swimming can be seen.

Thus the simplified dynamics are here seen to capture, predict, and correspond to complex behaviors of the higher-dimensional system, in the case of both near-boundary and bulk swimming, and hence the study of monoflagellate motion restricted to the given planes is evidenced to be remarkably descriptive of and pertinent to the details of the unconstrained motion of the swimmer. Understanding why the full dynamical system collapses onto the reduced system, and whether it does for other swimmers more generally, warrants detailed further study.
